# Prevalence of Vitamin D Deficiency Among Young Elite Soccer Players Living Above 55 Degrees North Latitude and Evaluation of the Effectiveness of Self-Used Preventive Methods

**DOI:** 10.1155/tsm2/2299710

**Published:** 2025-04-11

**Authors:** Bezuglov Eduard, Achkasov Evgeniy, Vinogradov Mikhail, Baranova Daria, Shurygin Vladimir, Rudiakova Elizaveta, Usmanova Elvira, Vakhidov Timur, Malyakin Georgiy, Ilsiuiar Anishchenko, Kapralova Elizaveta

**Affiliations:** ^1^Department of Sports Medicine and Medical Rehabilitation, Sechenov First Moscow State Medical University of the Ministry of Health of the Russia Federation, Moscow, Russia; ^2^High Performance Sports Laboratory, Sechenov First Moscow State Medical University, Moscow, Russia; ^3^Medical Department, PFC “CSKA”, Moscow, Russia; ^4^Department of Medical Rehabilitation, Odintsovo Regional Hospital, Odintsovo, Russia

**Keywords:** 25(OH)D, nutritional supplement, soccer, vitamin D, vitamin D deficiency, vitamin D insufficiency, young soccer players

## Abstract

The widespread prevalence of vitamin D deficiency among young elite soccer players living above 40° north latitude is a significant issue. Considering the adverse effects of vitamin D deficiency, it is crucial to investigate its prevalence and the effectiveness of self-used preventive strategies among high-risk groups. This study involved 209 young athletes (aged 7–18 years, mean age: 12.79 ± 3.04 years, weight: 50.11 ± 17.75 kg, height: 1.60 ± 0.19 m, and BMI: 18.69 ± 2.75) from a leading soccer academy, residing above 55° north latitude. Blood samples were collected in winter to analyze the total 25-hydroxyvitamin D (25(OH)D) levels using liquid chromatography-mass spectrometry. High prevalence of insufficiency (38.3%) and deficiency (26.8%) of 25(OH)D was identified. There were no significant differences in the severity of deficiency among different age groups (6–9 years, 10–14 years, and 15–18 years) or during growth spurts. The analysis of self-used preventive methods showed no significant differences between the compared groups (*p*=0.149). Vitamin D deficiency and insufficiency are widespread among young elite soccer players living above 55° north latitude and training indoors. The effectiveness of self-used preventive methods is considered low.

## 1. Introduction

Vitamin D deficiency among various populations is a significant global health problem, as its negative impact on multiple health aspects is well documented [1, 2]. This deficiency is prevalent among different population groups, including physically active individuals and athletes of varying levels [3–5].

The primary adverse effects of vitamin D deficiency are related to bone health, highlighting its crucial role in calcium–phosphorus metabolism [6–8].

In addition, vitamin D is essential for the immune [9], endocrine [10], and muscular systems [11, 12], affecting muscle strength, glucose metabolism, immunomodulatory activity of monocytes, and activation of T- and B-lymphocytes.

Given the importance of vitamin D for overall health, it is critical to address recognized risk factors for its deficiency and develop measures for timely diagnosis and safe correction. Risk factors for vitamin D deficiency include residing above 40° north latitude [13, 14], insufficient outdoor time [15, 16], and possibly intense physical activity [17, 18].

These risk factors significantly impact young athletes living above 55° north latitude and training indoors [19]. During winter, lower sunlight exposure and shorter daylight hours increase vulnerability to vitamin D deficiency [20].

Therefore, it is practically relevant to study the prevalence of vitamin D deficiency and evaluate the effectiveness of self-used preventive methods among young elite soccer players residing above 55° north latitude and training indoors during winter.

In this study, it was hypothesized that young elite soccer players living above 55° north latitude and training primarily indoors would have a high prevalence of vitamin D deficiency. It was also hypothesized that self-administered preventive measures, if implemented, would be insufficient to adequately address this deficiency, highlighting the need for targeted and structured intervention strategies.

## 2. Materials and Methods

### 2.1. Ethical Approval

The study was carried out in accordance with the Declaration of Helsinki. The protocol of the study was approved by the official Local Ethics Committee of the Sechenov First Moscow State University with the number 11–19 of 25 July 2019.

### 2.2. Subjects

The study involved 209 young soccer academy athletes aged 7–18 years (mean age: 12.79 ± 3.04 years, weight: 50.11 ± 17.75 kg, height: 1.60 ± 0.19 m, and BMI: 18.69 ± 2.75) residing above 55° north latitude, primarily training indoors or in the evening over the past three months. Participants were recruited from one of the country's leading soccer academies, ensuring a high level of athletic proficiency and a standardized training environment.

### 2.3. Laboratory Testing

During routine preseason medical examinations in February 2024, blood samples were collected from all subjects in the morning, fasting, between 8:00 and 10:00 a.m. from the cubital vein by an experienced technician. Athletes were instructed to rest the day before the blood draw.

Total 25(OH)D levels were measured using liquid chromatography-mass spectrometry (Agilent 1200 liquid chromatograph and AB Sciex 3200 MD mass detector). Concentrations were assessed through the total 25(OH)D level, the most suitable marker for the vitamin D status [21, 22].

Vitamin D status was classified as deficient (< 20 ng/mL), insufficient (20–30 ng/mL), and sufficient (> 30 ng/mL) [23]. All participants completed a food frequency and lifestyle questionnaire (FFLQ), adapted to local dietary preferences, previously used in high-methodological-quality studies to assess vitamin D intake [24]. The prevalence of preventive measures (self-supplementation, tanning bed use, and travel to higher UV index countries) was evaluated based on these responses. Skeletal age and growth phase were determined using the Sonic Bone ultrasound device (BAUSport, Israel).

Inclusion criteria: residing above 55° north latitude for the past 3 months, no missed training sessions due to injuries or illnesses in the month preceding the study, and informed consent from participants and their guardians.

Exclusion criteria: separate preseason medical examination from the main group and refusal to participate at any stage by participants or their guardians.

### 2.4. Statistical Analysis

Data were analyzed using Python (Version 3.8; Python Software Foundation, Wilmington, DE, USA) with Pandas for data processing and SciPy for statistical testing. Results were compiled and presented using Microsoft Excel (Microsoft Corporation, Redmond, WA, USA). Variables included plasma 25(OH)D concentration, age, height, weight, and BMI.

Normality of data distribution was assessed using the Shapiro–Wilk test. Depending on normality results, ANOVA was used for normally distributed data, the Kruskal–Wallis test for more than two groups with non-normal distribution, and the Mann–Whitney *U* test for two groups with non-normal distribution. Categorical data were analyzed using the chi-square test for independence to determine significant associations between serum 25(OH)D status and supplement use. Results were systematically organized in tables, displaying mean values with standard deviations (±), counts, and percentages for each group. Statistical significance was set at *p* < 0.05.

## 3. Results

### 3.1. Serum 25(OH)D Status of the Participants

Analysis revealed normal 25(OH)D levels in 34.9% and varying degrees of insufficiency in 65.1% of the participants. The mean serum 25(OH)D concentration was 27.45 ± 11.48 ng/mL. No significant differences in age, weight, and BMI were found between groups with different 25(OH)D status (*p* > 0.05). Participants with normal 25(OH)D levels had significantly higher mean concentrations (39.33 ± 10.31 ng/mL) compared with those with insufficient (24.87 ± 3.08 ng/mL) and deficient (15.64 ± 2.54 ng/mL) levels (*p* ≤ 0.001) (Table 1).

Stratification by age groups (6–9 years, 10–14 years, and 15–18 years) showed no significant differences in serum 25(OH)D concentrations, but there were statistically significant differences in all anthropometric data (Table 2).

No significant differences in serum 25(OH)D concentrations were found between athletes in and out of growth spurts (Table 3).

Self-reported vitamin D supplementation was used by 52.6% of the participants. There were no significant differences in serum 25(OH)D levels between those who used supplements and those who did not (*p*=0.149). Notably, in 84.2% of the cases (176 participants), athletes (or their representatives) did not know the exact dosage. When known, dosages ranged from 500 to 1500 IU/day. No significant association was found between supplement use and serum 25(OH)D levels among participants (*p*=1 for all categories) (Table 4).

The results of the study confirmed our hypothesis that vitamin D deficiency and insufficiency are highly prevalent in elite young elite soccer players who live above 55° north latitude and train indoors. Furthermore, the analysis showed that self-administered preventive measures were largely ineffective in addressing the problem, highlighting the need for more structured and evidence-based strategies to combat vitamin D deficiency in this vulnerable population.

## 4. Discussion

The findings show a high prevalence of vitamin D insufficiency among young elite soccer players: 38.3% had insufficient levels and 26.8% had deficient levels. This prevalence was consistent across all age groups (6–9, 10–14, and 15–18 years).

These results align with previous studies on young athletes residing in similar geographic regions (above 55° north latitude). For instance [25], found vitamin D insufficiency in 19.9% and deficiency in 22.9% of young soccer players during winter. The lower prevalence in that study could be attributed to its timing (December) compared with this study (February), reflecting a longer period of indoor training.

Other studies reported lower prevalence rates. For example, a study on young track and field athletes above 40° north latitude found deficiency in only 5.8% of the participants [26]. This difference may be due to the summer season and outdoor training for at least 3 months before the study.

It is well-established that vitamin D insufficiency is highly prevalent among young athletes living above 55° north latitude during winter [19, 25, 27]. Therefore, it is crucial to implement preventive measures, such as consuming vitamin D-rich and fortified foods [28], dietary supplements [29], using tanning beds [30], and spending time outdoors during peak sunlight hours (10:00 a.m.–3:00 p.m.) [29] with exposed skin without using sunscreen [31].

Foods rich in vitamin D include cod liver oil (8400 IU/100 g), marine fish (200–650 IU/100 g), oysters (320 IU/100 g), chicken eggs (125 IU/100 g), and mushrooms (75 IU/100 g) [32]. Participants' diets lacked these foods. In addition, their school schedule (9:00 a.m.–2:00 p.m.) limited their outdoor exposure during peak sunlight hours [33].

The only consciously used preventive method in this study was vitamin D supplementation, practiced by 52.6% of the participants. They used oral forms (drops and capsules) but mostly did not know the exact dosage (84.2%). When known, the dosage ranged from 500 to 1500 IU/day.

The effectiveness of self-used supplementation was low: the prevalence of serum 25(OH)D insufficiency and deficiency was similar in both supplement-using and nonusing groups (insufficiency: 35.5% and 41.4% and deficiency: 39.1% and 30.3%, respectively; *p* > 0.05).

This study is the first to report serum 25(OH)D status among young athletes in growth spurts. This period involves accelerated body size increase, theoretically requiring higher vitamin D intake and other substances vital for calcium-phosphorus metabolism [34].

However, the prevalence of 25(OH)D insufficiency was similar among athletes in and out of growth spurts. Nevertheless, widespread insufficiency and deficiency in growth spurts could negatively impact bone health, potentially leading to conditions like osteochondropathies. A previous study by Smida et al. showed that healthy children with Osgood–Schlatter disease, associated with growth spurts, had a higher prevalence of vitamin D deficiency [35].

Study limitations include the methodology for collecting dietary information. Participants filled out an FFLQ, marking vitamin D-rich foods consumed in the past 3 months. This method may be less accurate than maintaining a food diary, potentially due to participants' forgetfulness.

### 4.1. Clinical Implications

Practitioners should focus on raising awareness among athletes, their parents, and coaches about maintaining reference vitamin D levels, familiarizing them with strategies to increase serum vitamin D, and developing educational programs for these groups.

### 4.2. Limitations

The study did not collect detailed information on several potential covariates that could influence vitamin D levels, such as the amount of sunlight exposure, specific dietary intake of vitamin D-rich foods, and training intensity. The absence of this data limits our ability to adjust for these factors in the analysis, which could potentially confound the relationship between self-reported vitamin D supplementation and serum 25(OH)D levels. Future research should incorporate measures of sunlight exposure, detailed dietary logs, and training details.

## 5. Conclusion

Vitamin D insufficiency and deficiency are widespread among young elite soccer players living above 55° north latitude during winter, and growth spurts do not increase its prevalence. The only preventive method used was vitamin D supplementation, whose effectiveness was low.

## Figures and Tables

**Table 1:** Vitamin D status and associated metrics.

Group	Sample size (*n* (%))	Mean 25(OH)D (ng/mL)	Age (years)	Height (m)	Weight (kg)	BMI (kg/m^2^)
Total	209 (100.0%)	27.45 ± 11.48	12.79 ± 3.04	1.60 ± 0.19	50.11 ± 17.75	18.69 ± 2.75
Sufficient 25(OH)D	73 (34.9%)	39.33 ± 10.31	13.19 ± 2.83	1.64 ± 0.17	52.51 ± 16.27	18.92 ± 2.56
Insufficient 25(OH)D	80 (38.3%)	24.87 ± 3.08	12.45 ± 3.26	1.56 ± 0.19	46.71 ± 17.30	18.30 ± 2.83
Deficient 25(OH)D	56 (26.8%)	15.64 ± 2.54	12.77 ± 2.96	1.62 ± 0.21	51.89 ± 19.68	18.95 ± 2.84
*p* value		*p* ≤ 0.001	*p*=0.376	*p*=0.040	*p*=0.086	*p*=0.240

**Table 2:** Comparison of anthropometric data and serum 25(OH)D concentrations by age groups.

Group	Sample size (*n* (%))	Mean 25(OH)D (ng/mL)	Age (years)	Height (m)	Weight (kg)	BMI (kg/m^2^)
Total	209 (100.0%)	27.45 ± 11.48	12.82 ± 3.02	1.60 ± 0.19	50.11 ± 17.75	18.69 ± 2.75
6–9 years	37 (17.70%)	25.61 ± 8.77	8.36 ± 0.92	1.34 ± 0.08	28.84 ± 5.01	15.97 ± 1.20
10–14 years	107 (51.20%)	27.28 ± 10.00	12.18 ± 1.49	1.57 ± 0.12	44.92 ± 11.29	17.89 ± 2.16
15–18 years	65 (31.10%)	28.77 ± 14.68	16.33 ± 1.07	1.81 ± 0.07	70.44 ± 8.70	21.50 ± 1.62
*p* value		*p*=0.637	*p* ≤ 0.001	*p* ≤ 0.001	*p* ≤ 0.001	*p* ≤ 0.001

**Table 3:** Anthropometric data and serum 25(OH)D concentrations by growth phase.

Group	Sample size (*n* (%))	Mean 25(OH)D (ng/mL)	Age (years)	Height (m)	Weight (kg)	BMI (kg/m^2^)
In growth spurt	47 (22.49%)	28.52 ± 9.51	13.46 ± 1.16	1.65 ± 0.06	52.78 ± 8.18	19.23 ± 2.14
Out of growth spurt	162 (77.51%)	27.13 ± 12.00	12.60 ± 3.37	1.59 ± 0.21	49.33 ± 19.64	18.53 ± 2.88
p value		*p*=0.160	*p*=0.089	*p*=0.078	*p*=0.084	*p*=0.070

**Table 4:** Effect of supplementation on serum 25(OH)D concentrations and antropometrics

	Using supplementation	Not using supplementation	*p* value
Sample size (*n* (%))	110 (52.63%)	99 (47.37%)	
Mean 25(OH)D (ng/mL)	28.06 ± 10.63	26.76 ± 12.37	*p*=0.149
Age (years)	12.87 ± 2.79	12.71 ± 3.30	*p*=0.645
Height (m)	1.61 ± 0.19	1.59 ± 0.19	*p*=0.427
Weight (kg)	50.58 ± 17.11	49.60 ± 18.51	*p*=0.579
BMI (kg/m^2^)	18.70 ± 2.65	18.68 ± 2.85	*p*=0.870
Sufficient 25(OH)D (*n* (%))	28 (25.5%)	28 (28.3%)	1
Insufficient 25(OH)D (*n* (%))	39 (35.5%)	41 (41.4%)	1
Deficient 25(OH)D (*n* (%))	43 (39.1%)	30 (30.3%)	1

## Data Availability

The data that support the findings of this study are available from the corresponding author upon reasonable request.
